# Supplementary material for: Enhancing tandem mass spectrometry-based metabolite annotation with online chemical labeling
Source: Nat Commun. 2025 Jul 26;16:6911. doi: 10.1038/s41467-025-61240-z (PMC12297239; doi:10.1038/s41467-025-61240-z)
Supplement: Supplementary file 1 — Supplementary Information [file 41467_2025_61240_MOESM1_ESM.pdf]

Supplemental Information

Enhancing tandem mass spectrometry-based metabolite annotation with online chemical labeling

Giovanni Andrea Vitale[1,*], Shu-Ning Xia[1,*], Kai Dührkop[2,3], Mohammad Reza Zare Shahneh[4], Heike Brötz-Oesterhelt[1,5,6], Yvonne Mast[6,7], Corinna Brungs[8,9], Sebastian Böcker[2], Robin Schmid[8,10], Mingxun Wang[4], Chambers C. Hughes[1,5,6,#], Daniel Petras[6,11,#]

1. Department of Microbial Bioactive Compounds, Interfaculty Institute of Microbiology and Infection Medicine (IMIT), University of Tübingen, Tübingen, Germany
2. Chair for Bioinformatics, Institute for Computer Science, Friedrich Schiller University Jena, Jena, Germany
3. Bright Giant GmbH, Jena, Germany
4. Department of Computer Science, University of California Riverside, Riverside, CA, USA
5. German Center for Infection Research (DZIF), Partner Site Tübingen, Tübingen, Germany
6. Cluster of Excellence EXC 2124: Controlling Microbes to Fight Infection, University of Tübingen, Tübingen, Germany
7. Department Bioresources for Bioeconomy and Health Research, Leibniz Institute DSMZ - German Collection of Microorganisms and Cell Cultures, Braunschweig, Germany
8. Institute of Organic Chemistry and Biochemistry of the Czech Academy of Sciences, Prague, Czech Republic
9. Division of Pharmacognosy, Department of Pharmaceutical Sciences, Faculty of Life Sciences, University of Vienna, Vienna, Austria
10. mzio GmbH, Bremen, Germany
11. Department of Biochemistry, University of California Riverside, Riverside, CA, USA

* Contributed equally
# Correspondence: chambers.hughes@uni-tuebingen.de or dpetras@ucr.edu

Table of Content

Supplementary Figure 1 Derivatization reaction schemes
Supplementary Figure 2 Reaction A titration plots
Supplementary Figure 3 Reaction B titration plots
Supplementary Figure 4 Reaction C titration plots
Supplementary Figure 5 Extracted Ion Chromatograms (EIC) of the standards reacting with cysteine (Reaction A)
Supplementary Figure 6 Extracted Ion Chromatograms (EIC) of the standards reacting with AQC (Reaction B)
Supplementary Figure 7 Extracted Ion Chromatograms (EIC) of the standards reacting with hydroxylamine (Reaction C)
Supplementary Figure 8 Mass spectrum and structures of observed L-cysteine ion forms
Supplementary Figure 9 Online Reactivity Module interface in mzmine and parameters explanation
Supplementary Figure 10 Feature visualization in mzmine Online Reactivity Module
Supplementary Figure 11 Metadata in mzmine
Supplementary Figure 12 Impact of MCheM on Top 1 and Top 5 results from GNPS2 analog search for the experimental dataset
Supplementary Figure 13 Impact of MCheM on Top 10 analog search on GNPS2 for the experimental dataset
Supplementary Figure 14 Impact of MCheM on Top 1 and Top 5 analog search on GNPS2 for the CANOPUS dataset
Supplementary Figure 15 Impact of MCheM on Top 10 analog search on GNPS2 for the CANOPUS dataset
Supplementary Figure 16 Impact of MCheM on the average ranking of the most similar structure
Supplementary Figure 17 Oxazolomycin B MIBiG reference sequence
Supplementary Figure 18 MS/MS spectrum of putative oxazolomycin D (ID 1911) with its diagnostic fragments

Supplementary Figure 19 MS/MS spectrum of oxazolomycin D derivative with *m/z* 716.3754 (ID 1562) with its diagnostic fragments
Supplementary Figure 20 MS/MS spectrum of oxazolomycin D derivative with *m/z* 714.3598 (ID 1661) with its diagnostic fragments
Supplementary Figure 21 MS/MS spectrum of 7-glycosyl oxazolomycin D (ID 1569) with its diagnostic fragments.
Supplementary Figure 22 ^{1}H NMR (DMSO-d_6, 700 MHz) of 7-glycosyl oxazolomycin D
Supplementary Figure 23 ^{13}C NMR (DMSO-d_6, 700 MHz) of 7-glycosyl oxazolomycin D
Supplementary Figure 24 COSY NMR (DMSO-d_6, 700 MHz) of 7-glycosyl oxazolomycin D
Supplementary Figure 25 HSQC NMR (DMSO-d_6, 700 MHz) of 7-glycosyl oxazolomycin D
Supplementary Figure 26 HMBC NMR (DMSO-d_6, 700 MHz) of 7-glycosyl oxazolomycin D
Supplementary Figure 27 NOESY NMR (DMSO-d_6, 700 MHz) of 7-glycosyl oxazolomycin D

Supplementary Table 1 NMR spectral data for 7-glycosyl oxazolomycin D in DMSO-d_6 at 700 MHz
Supplementary Table 2 Datasets produced in this study

Additional Data Tables available as .csv, .tsv or . .xlsx files

**Supplemental Data 1**	Table with all tested Natural Product Standards
**Supplemental Data 2**	Table with MCheM reactions SMARTS
**Source Data**	SIRIUS and GNPS2 Source Date for Figure 3

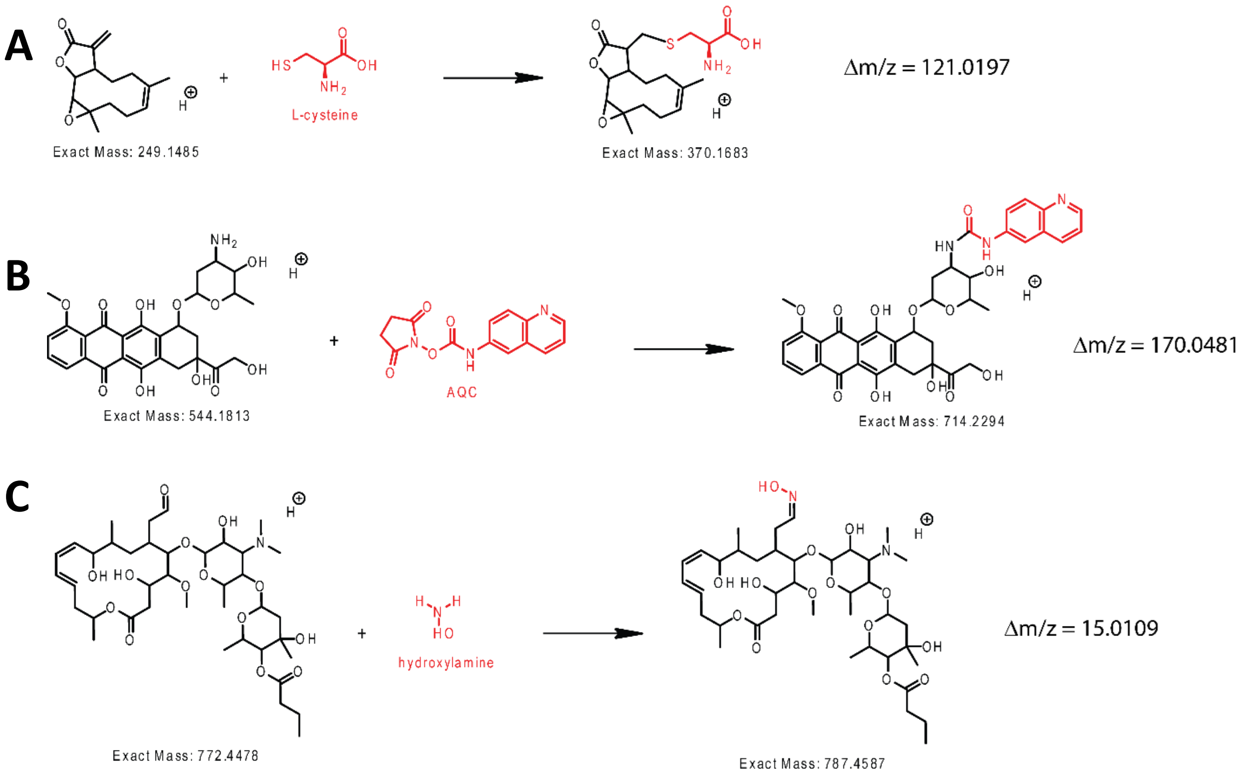

Supplementary Figure 1. Derivatization reaction schemes. Examples of the three derivatization reactions described in this manuscript with precursor and product *m/z* values and the corresponding expected mass shift of the [M+H]+ form. A) reaction A performed on parthenolide, B) reaction B performed on doxorubicin, and C) reaction C performed on kitasamycin with respective products.

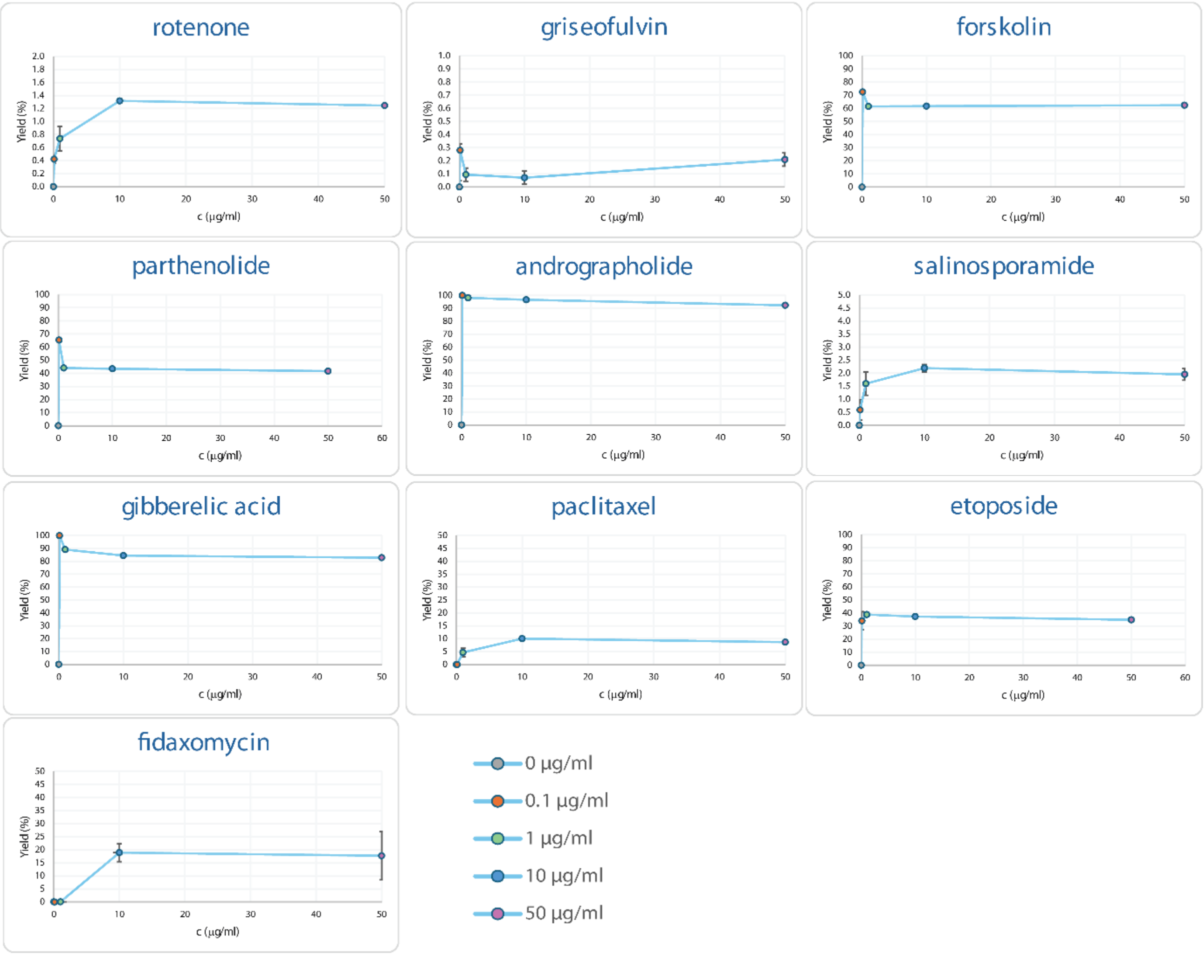

Supplementary Figure 2. Reaction A titration plots. Titration plots of the 32 standards reacting with L-cysteine 0.1 mM at different substrate concentrations as shown in the color chart. On the Y axis, it is reported the yield calculated on the [M+H]+ ions of the educts and products (except for fidaxomycin where [M+Na]+ (educt)/ [M+H]+(product) correlation was detected), while on the X axis the concentration in µg/ml of each standard in the mix. Error bars were drawn using the standard deviation between the two measurements.

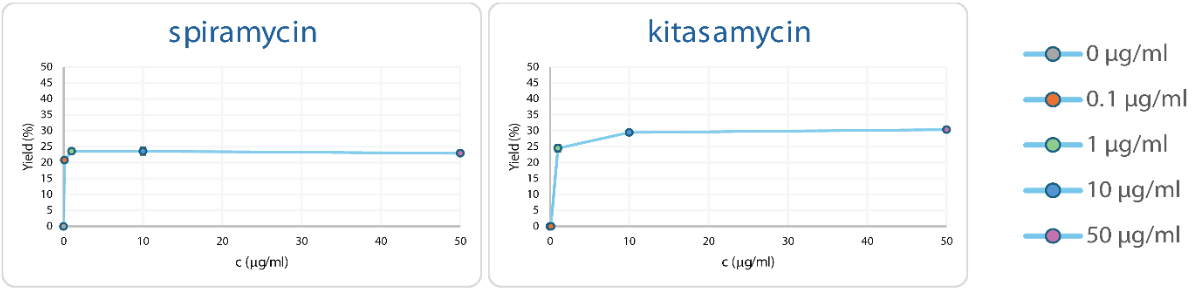

Supplementary Figure 3. Reaction B titration plots. Titration plots of 32 mix molecules reacting with hydroxylamine 10 mM at different concentrations (50, 10, 1 and 0.1 μg/ml). On the Y axis, it is reported the yield calculated on the [M+H]+ ions of the unmodified and the derivatized molecule, while on the X axis the concentration in μg/ml of each metabolite in the mix.

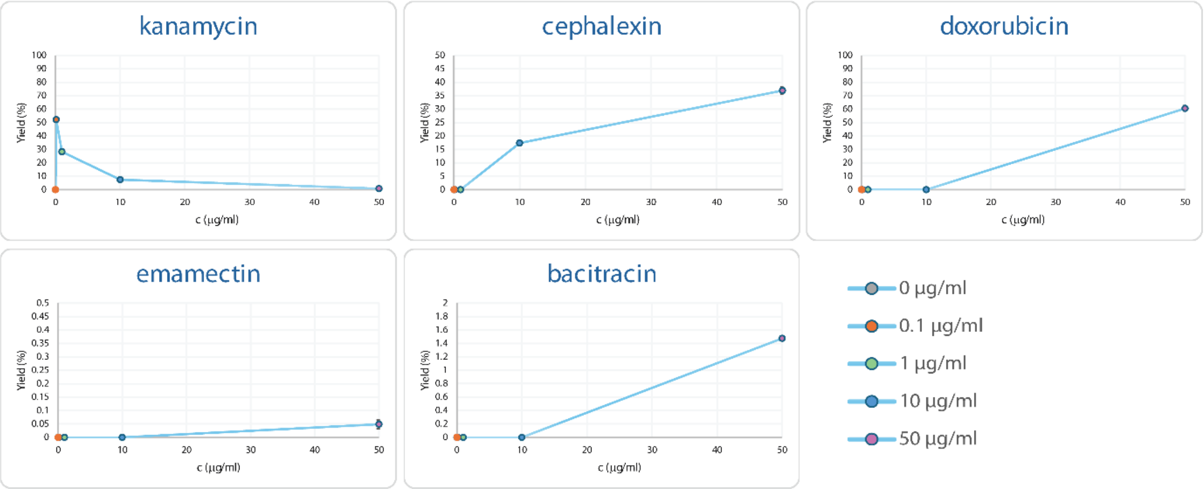

Supplementary Figure 4. Reaction C titration plots. Titration plots of 32 mix molecules reacting AQC 100 mM at different substrate concentrations as shown in the color chart. On the Y axis, it is reported the yield calculated on the $[M+H]^+$ ions of the educts and products (except for cephalexin where [M+Na]+ (educt)/ [M+H]+(product) correlation was detected), while on the X axis the concentration in µg/ml of each standard in the mix. Error bars were calculated using the standard deviation between the two measurements.

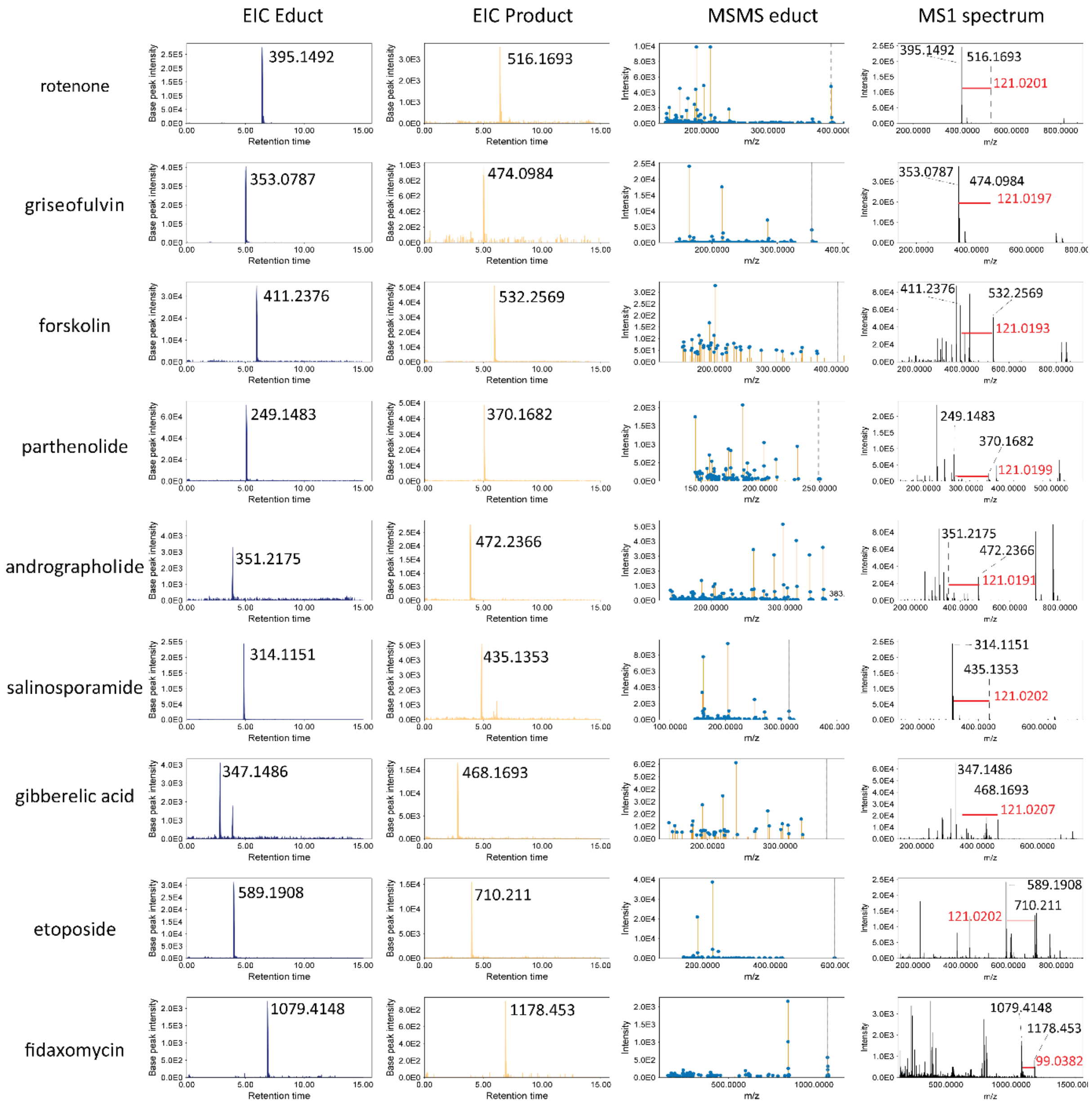

Supplementary Figure 5. Extracted Ion Chromatograms (EIC) of the standards reacting with cysteine (Reaction A). In the 32 standards mixture, EICs of respective products are shown appearing at the same retention time in the treated sample, MS/MS spectrum of the educts, and MS1 spectrum of the educts/products pairs observed at the same retention time in the treated samples, and connected via the peculiar reaction Δ*m/z* through the Online Reactivity. For all the molecules the reactivity was detected via the correlation between precursor and product [M+H]+ ions (calcld. Δ*m/z* = 121.0197), except for fidaxomycin (at the bottom) where [M+Na]+ (educt)/ [M+H]+(product) correlation was detected (calcld. Δ*m/z* = 99.03781).

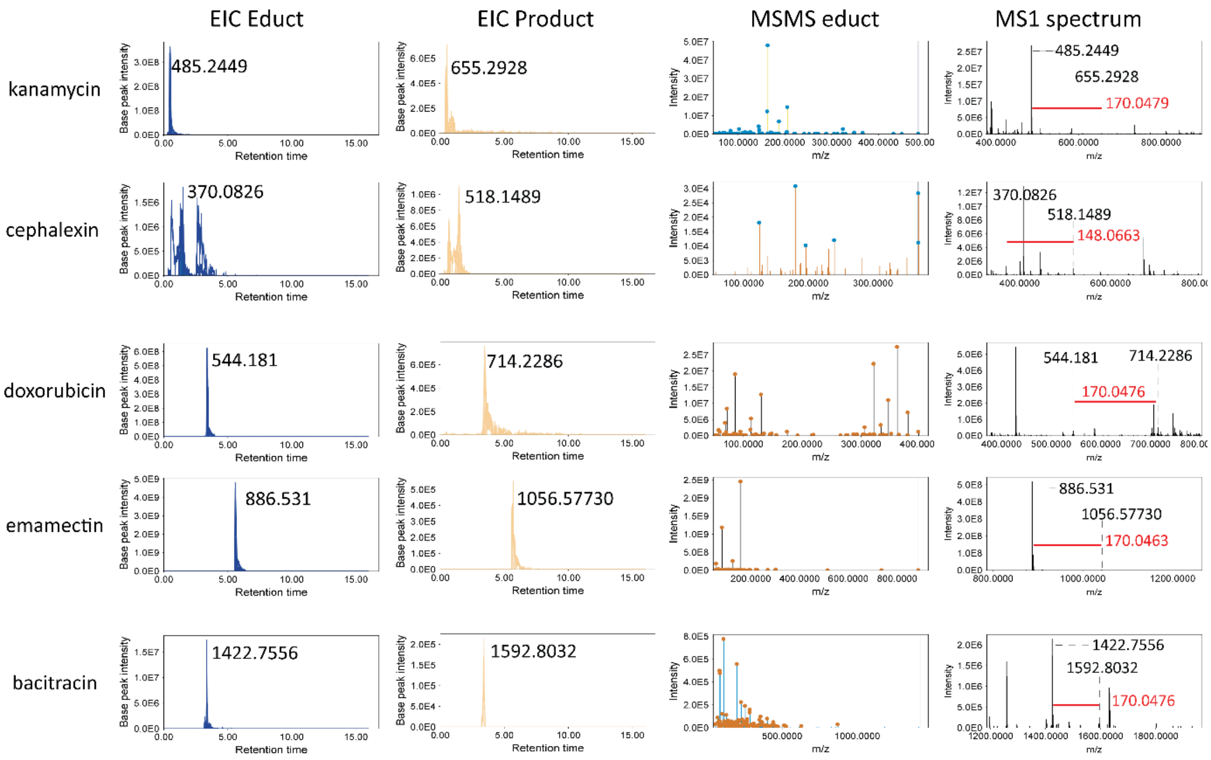

Supplementary Figure 6. Extracted Ion Chromatograms (EIC) of the standards reacting with AQC (Reaction B). In the 32 standards mixture, respective products EICs are shown, appearing at the same RT, MSMS spectrum of the educts, and MS1 spectrum of the educts/products pairs observed at the same retention time in the treated samples and connected via the peculiar reaction Δ*m/z* through the Online Reactivity. For all the molecules the reactivity was detected thanks to the correlation between educt and product [M+H]+ ions (calcld. Δ*m/z* = 170.0481), except for cephalexin where [M+Na]+ (educt)/ [M+H]+(product) correlation was detected (calcld. Δ*m/z* = 148.0661).

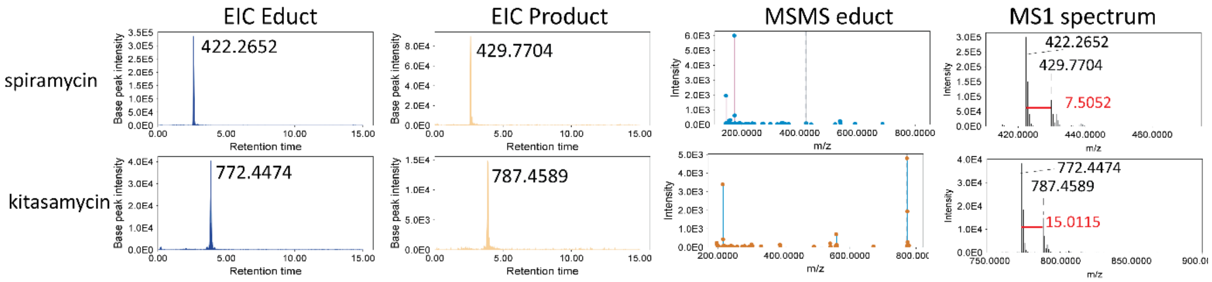

Supplementary Figure 7. Extracted Ion Chromatograms (EIC) of the standards reacting with hydroxylamine (Reaction C). From the 32 standards mixture, respective EICs are shown that appeared at the same RT, MS/MS spectrum of the educts, and MS1 spectrum of the educts/products pairs observed at the same retention time in the treated samples, and connected via the peculiar reaction Δ*m/z* through the Online Reactivity. For kitasamycin the reactivity was detected thanks to the correlation between educt and product [M+H]+ ions (calcld. Δ*m/z* = 15.0109), for spiramycin the correlation between the two [M+2H]2+ was detected (calcld. Δ*m/z* = 7.5054).

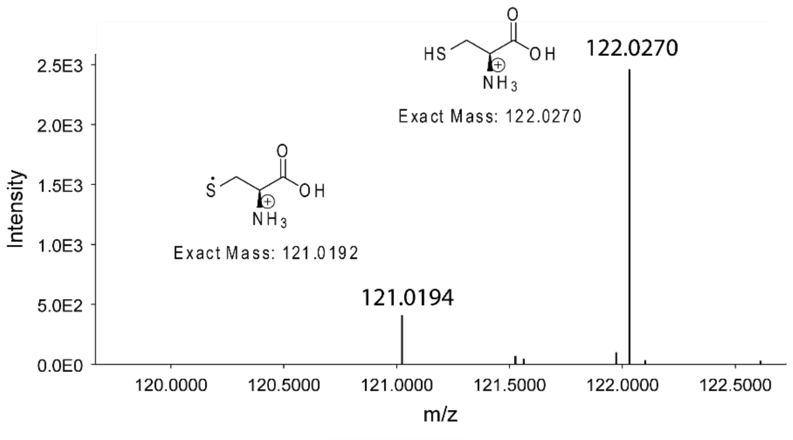

Supplementary Figure 8. Mass spectrum and structures of observed L-cysteine ion forms. Mass spectrum and structures of cysteine cation radical [M+H]·+ and protonated [M+H]+ forms, both were observed when during blank (MeOH) run with a continuous post-column injection of L-cysteine 0.1 mM (*m/z* range 100-300). MeOH without post-column injection of L-cysteine was also run, and none of the two ions was observed.

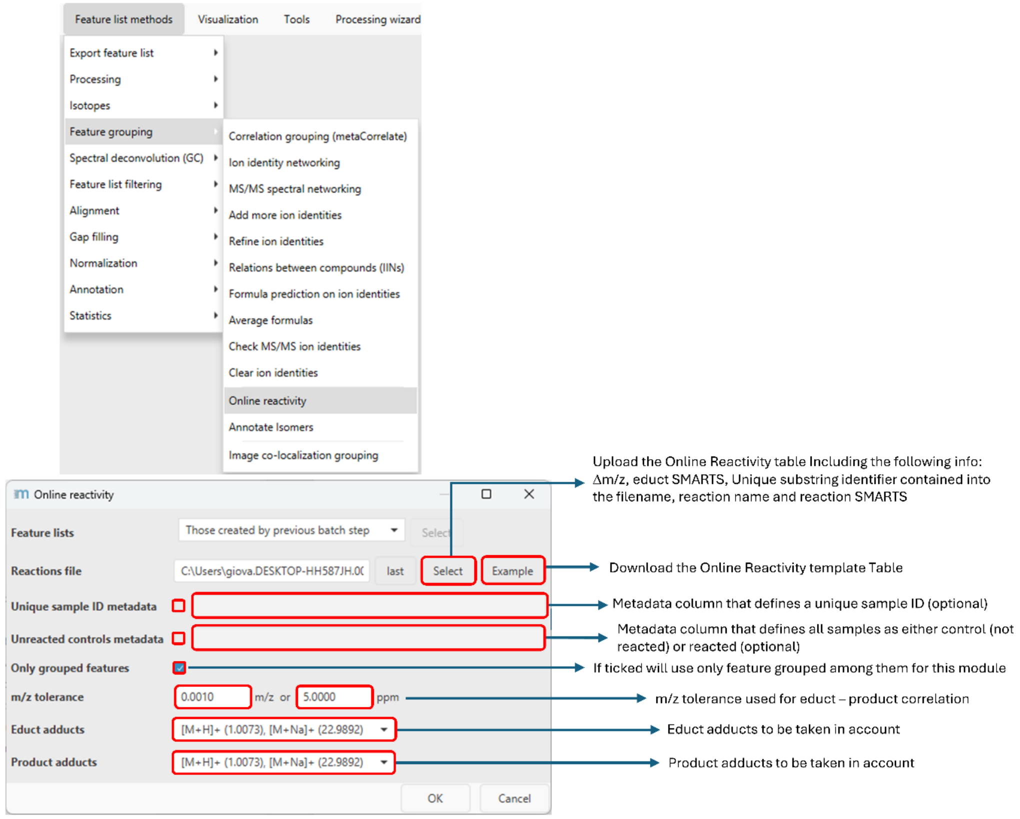

Supplementary Figure 9. Online Reactivity Module interface in mzmine and parameters explanation. Visualization of the Online Reactivity module of Mzmine4 built to automatically connect educt and products during an online reaction based on the typical D*m/z*. By clicking on the example button a template of the input table with the essential parameters for this module is provided, here the different reaction features are defined (I.e. Δ*m/z*, educt SMARTS, reaction SMARTS). If ticked the “Unique sample ID” and the “Reaction sample Type” can be used. The user can make use of the correlation produced by the meta correlation module if run before this one by ticking “Only grouped features”, moreover he can define the *m/z* tolerance and the allowed adducts for both educt and products.

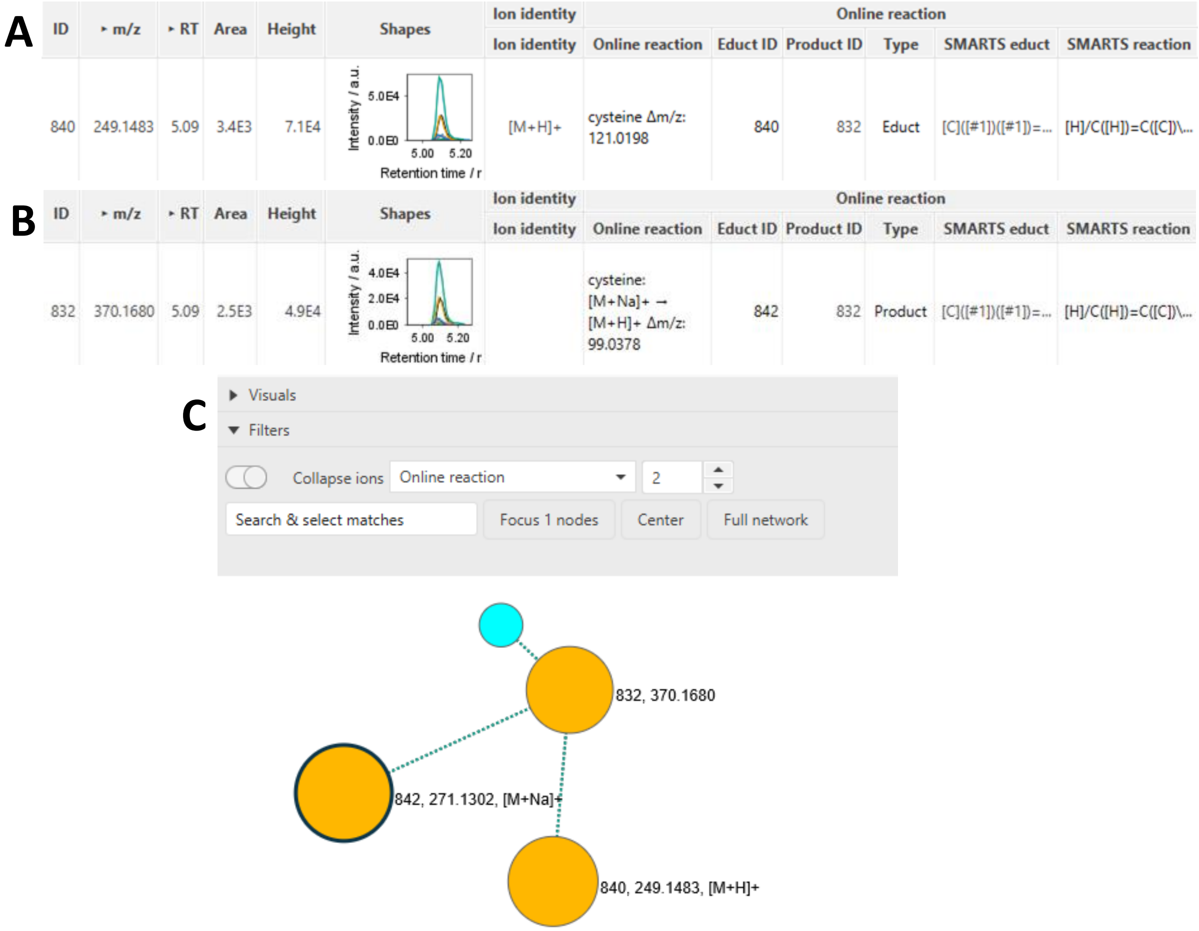

Supplementary Figure 10. Feature visualization in mzmine Online Reactivity Module. In the feature list, it is possible to activate the visualization of the online reaction attributes, such as the observed online reaction (including the ions involved) with the respective Δ*m/z*, educt, and product IDs, the status of the feature we are observing (educt or product) the putative educt substructure or the entire reaction (in the form of SMARTS). Here are shown as an example A) parthenolide [M+H]+ ion row and B) parthenolide derivatization product [M+H]+ with cysteine row, automatically connected by the Online reactivity module. By clicking on the feature, it is possible to visualize the relative networks where the cyan node represents the reaction product. C) Reactivity network of parthenolide.

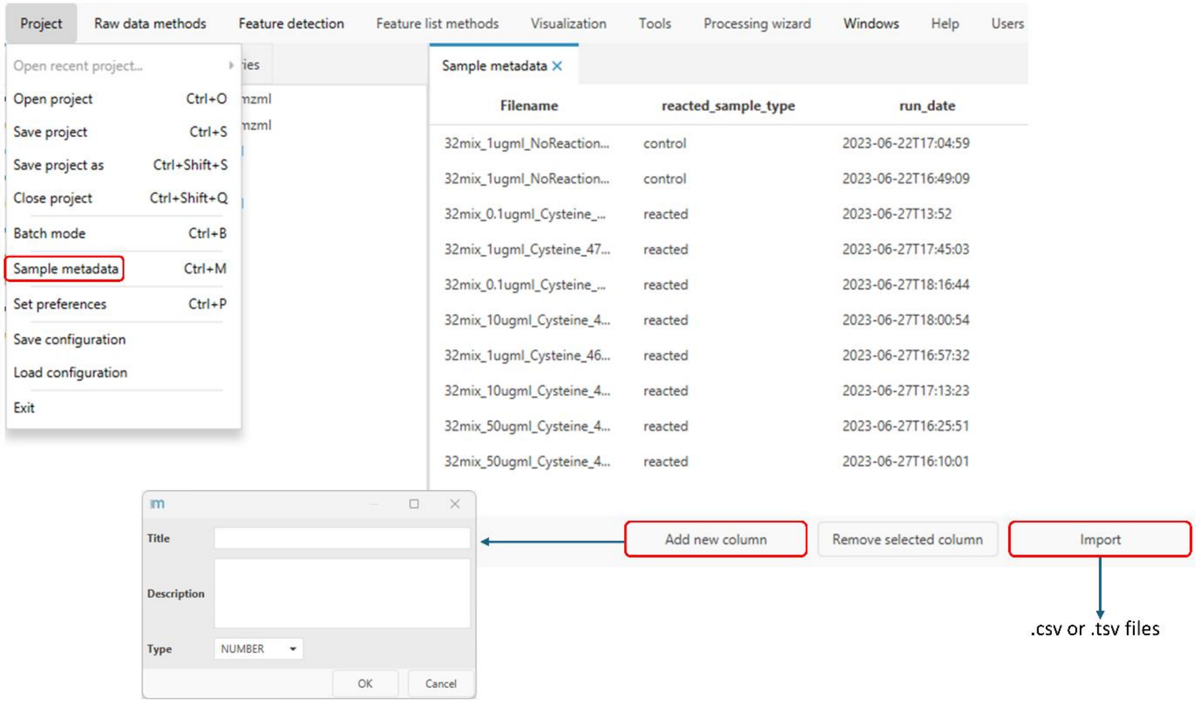

Supplementary Figure 11. Metadata in mzmine. Once a dataset is uploaded a simple Sample metadata table is automatically created and can be accessed by clicking on Project/Sample metadata. This metadata file originally contains the Filename and the run data columns, however, it is possible to either import a metadata table prepared offline in the .csv and .tsv format or to manually add additional columns.

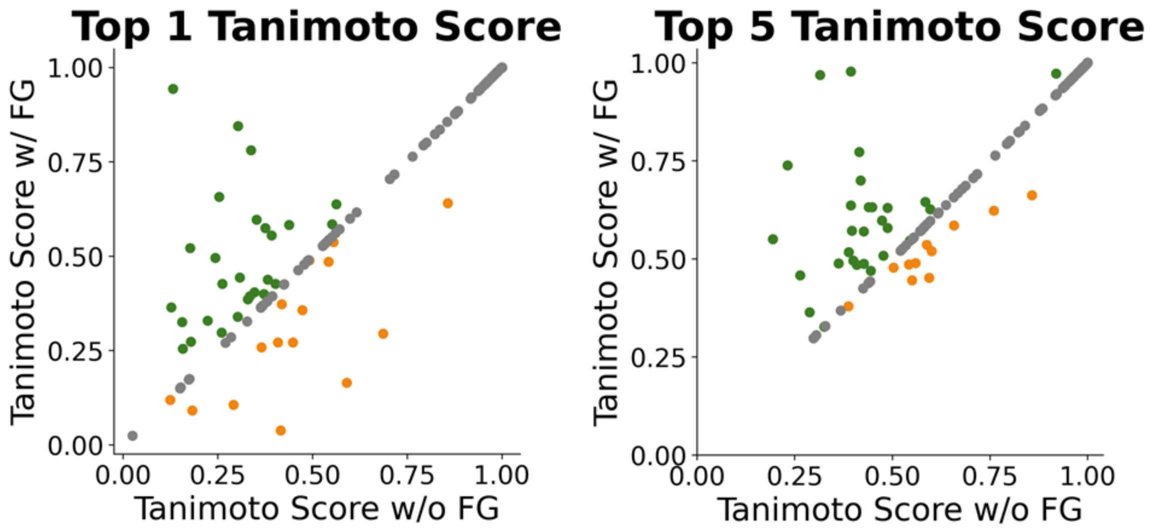

Supplementary Figure 12. Impact of MCheM on Top 1 and Top 5 results from GNPS2 analog search for the experimental dataset. The MCheM method improved the structural similarity of the Top 1, Top 5 analogs as reflected by the improved Tanimoto scores (green dots on the scatter plots), while a lower number of worsened features were retrieved (orange dots on the scatter plots), leading to an improved average Tanimoto score after FG-filtering compared with not filtered data (violin plots Figure 2 in the main text).

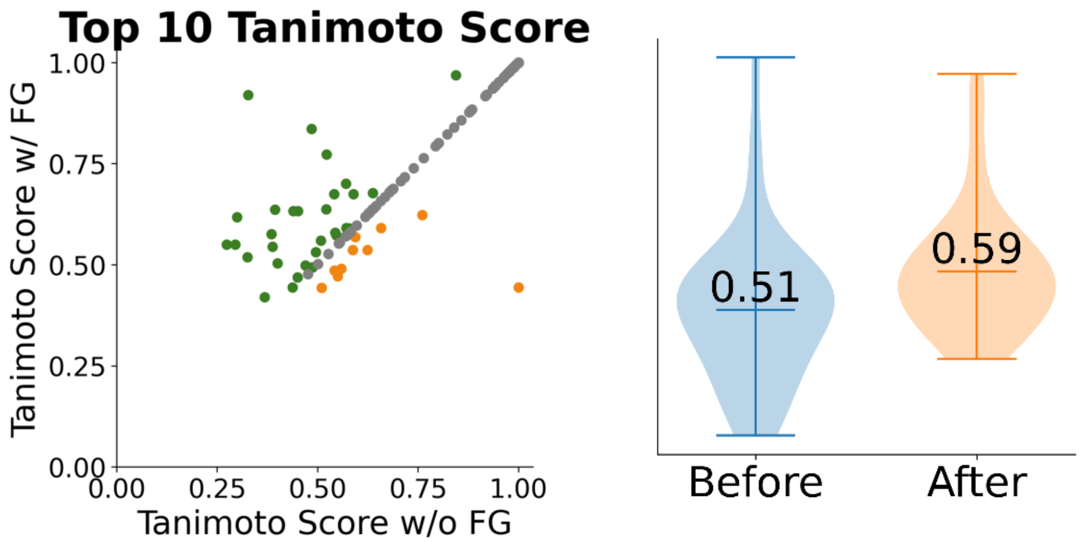

Supplementary Figure 13. Impact of MCheM on Top 10 analog search on GNPS2 for the experimental dataset. The MCheM method improved the similarity of Top 10 analogs with respect to the actual structure, as shown by the improved features reported as green dots in the scatter plot, while worsened annotations are reported as orange dots. Overall, MCheM showed an improvement in the average Tanimoto similarity score when compared to the classic annotation as depicted in the violin plot with the mean value going from 0.51 to 0.59. Datapoints from the scatter plots are visualized in the violine plots. Error bars indicate the spread of data and the center line indicates the mean.

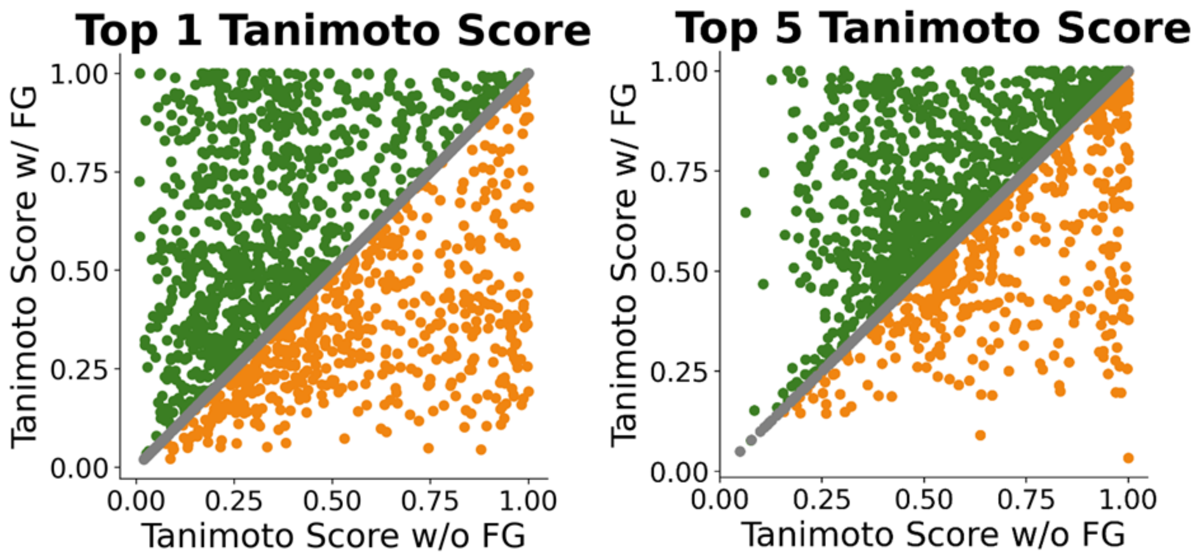

Supplementary Figure 14. Impact of MCheM on Top 1 and Top 5 analog search on GNPS2 for the CANOPUS dataset. The MCheM method improved the structural similarity of the Top 1, Top 5 analogs as reflected by the improved Tanimoto scores (green dots on the scatter plots), while a lower number of worsened features were retrieved (orange dots on the scatter plots), leading to an improved average Tanimoto score after FG-filtering compared with not filtered data violin plots Figure 2 in the main text).

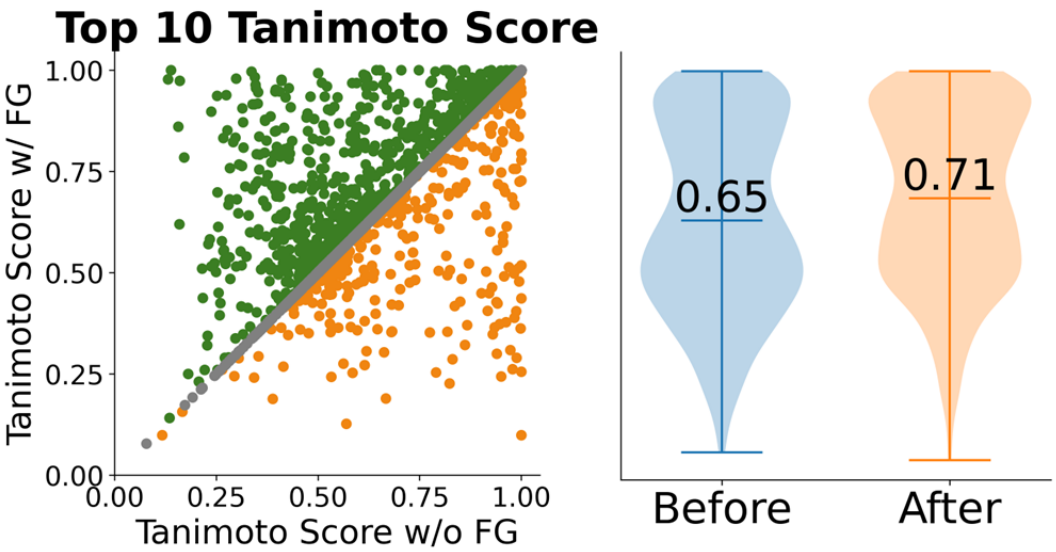

Supplementary Figure 15. Impact of MCheM on Top 10 analog search on GNPS2 for the CANOPUS dataset. The MCheM method improved the similarity of Top 10 analogs with respect to the actual structure, as shown by the improved features reported as green dots in the scatter plot, while worsened annotations are reported as orange dots. Overall, MCheM showed an improvement in the average Tanimoto similarity score when compared with the original annotation as depicted in the violin plot with mean value going from 0.65 to 0.71. Datapoints from the scatter plots are visualized in the violine plots. Error bars indicate the spread of data and the center line indicates the mean.

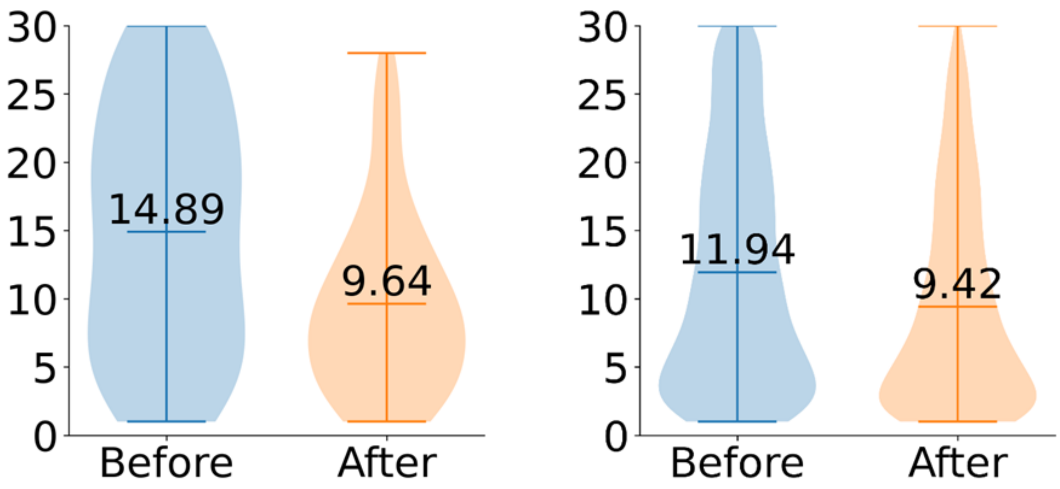

Supplementary Figure 16. Impact of MCheM on the average ranking of the most similar structure. The MCheM method substantially improved the average ranking of the most similar structure (orange violin) compared with the classic data (blue violin) both in the experimental (left plot) and CANOPUS (right plot) datasets. The mean values improved from 14.89 to 9.64 and from 11.94 to 9.42, and the shape also became narrower at the bottom for both datasets, which means a greater concentration of values closer to the 1st position. Error bars indicate the spread of data and the center line indicates the mean.

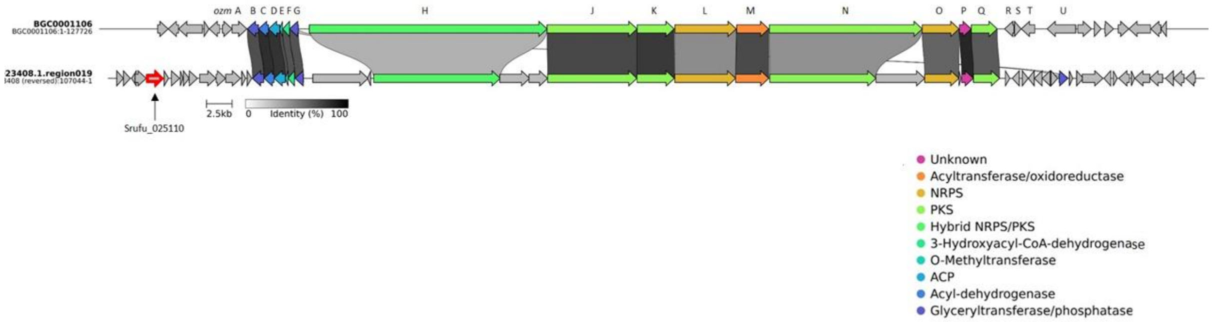

Supplementary Figure 17. Oxazolomycin B MIBiG reference sequence. BGC0001106 from *Streptomyces albus* JA3453 with *ozm* biosynthetic genes indicated (above) and cluster region 1.19 from *Streptomyces libani* subsp. *rufus* DSM 41230 (below). Glycosyltransferase gene *srufu*_25110 is highlighted in red. Gene cluster comparison was generated with clinker (Gilchrist et al., 2021).

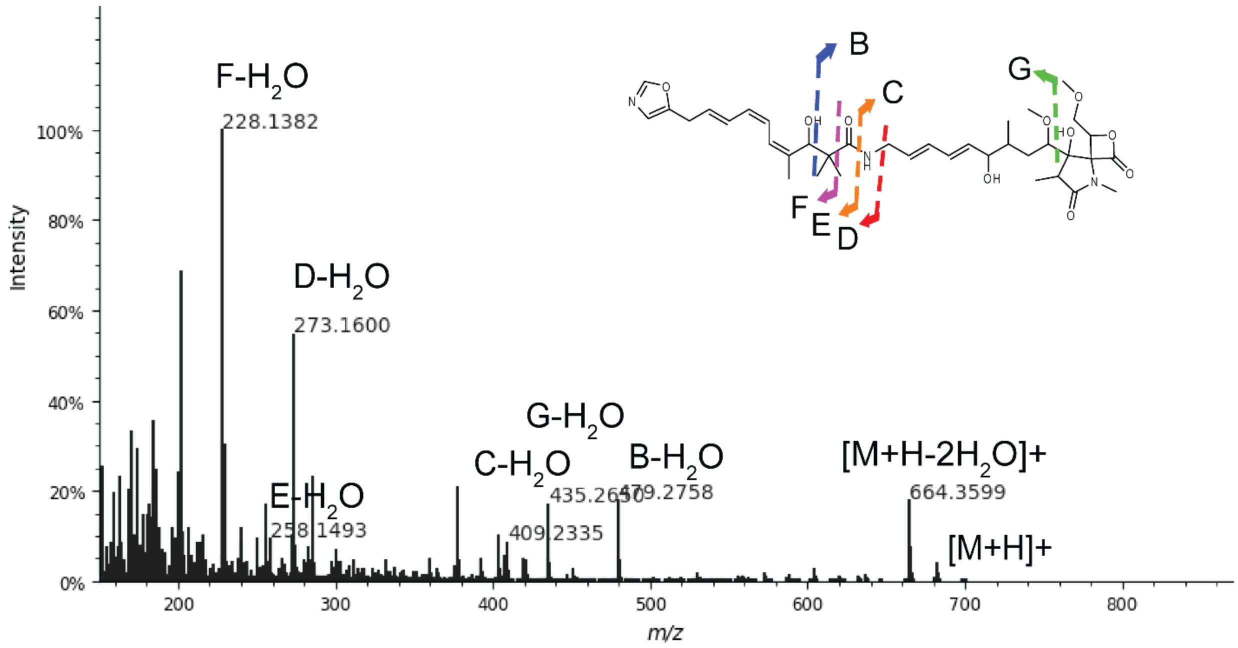

Supplementary Figure 18. MS/MS spectrum of putative oxazolomycin D (ID 1911) with its diagnostic fragments.

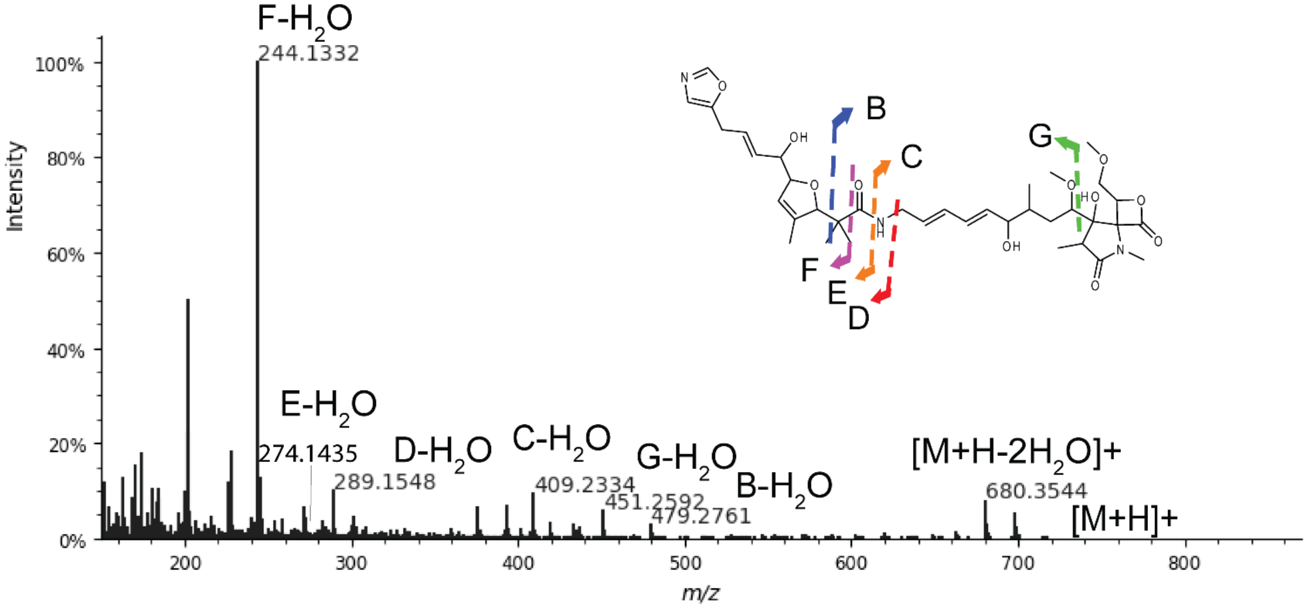

Supplementary Figure 19. MS/MS spectrum of oxazolomycin D derivative with *m/z* 716.3754 (ID 1562) with its diagnostic fragments. Unshifted C and B fragments allowed us to predict the modification on the left part of the molecule, as confirmed by a shift of F, E and D fragments.

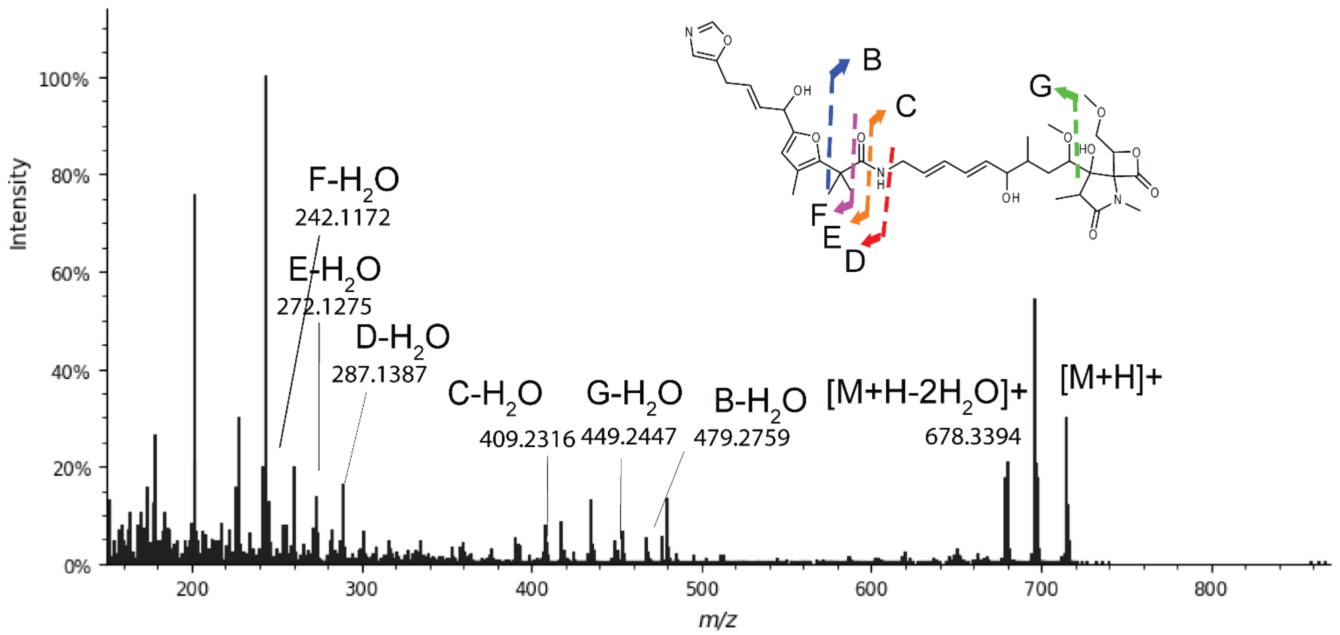

Supplementary Figure 20. MS/MS spectrum of oxazolomycin D derivative with *m/z* 714.3598 (ID 1661) with its diagnostic fragments. Unshifted C and B fragments allowed us to predict the modification on the left part of the molecule, as confirmed by a shift of F, E and D fragments.

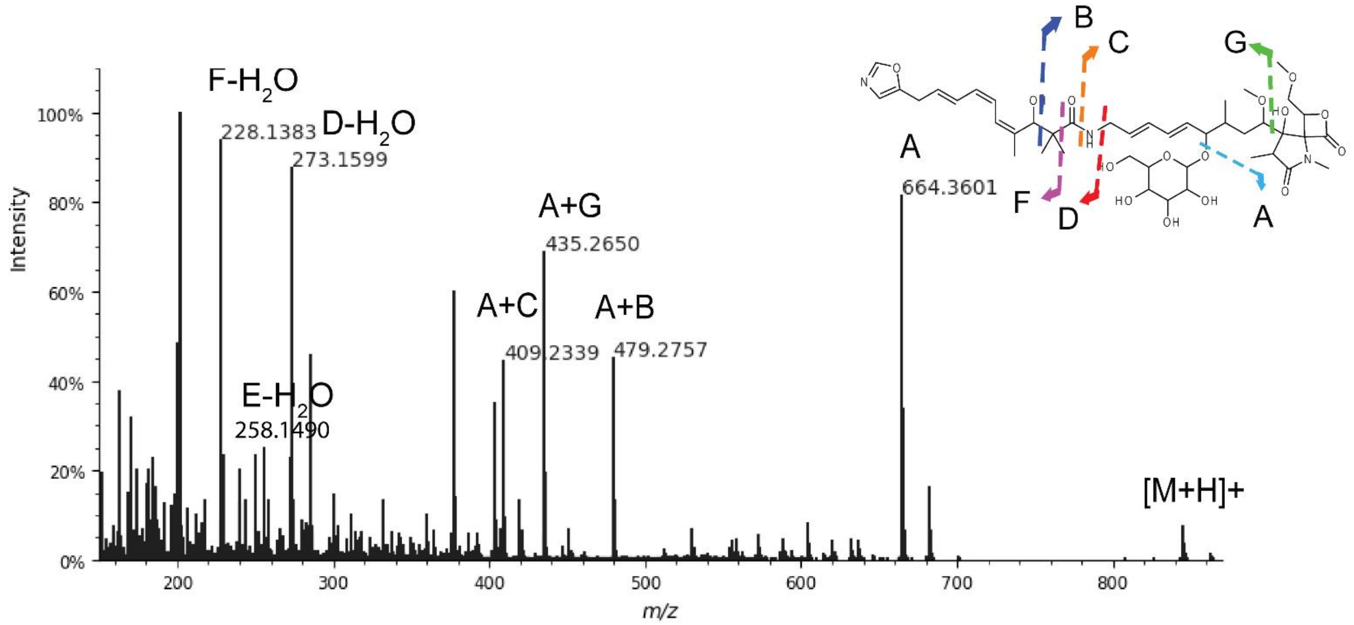

Supplementary Figure 21. MS/MS spectrum of 7-glycosyl oxazolomycin D (ID 1569) with its diagnostic fragments.

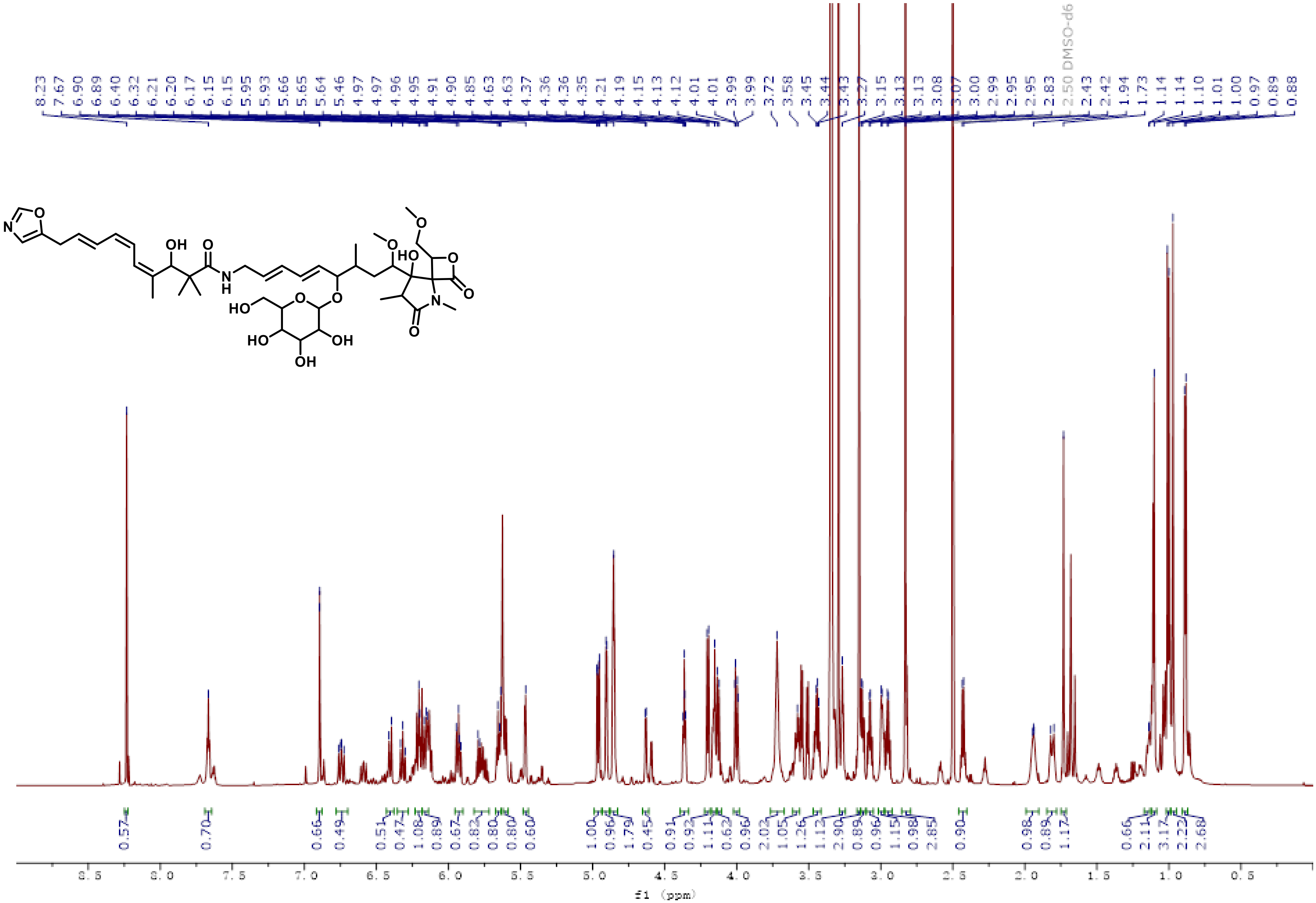

Supplementary Figure 22. ^{1}H NMR (DMSO-d_6, 700 MHz) of 7-glycosyl oxazolomycin D

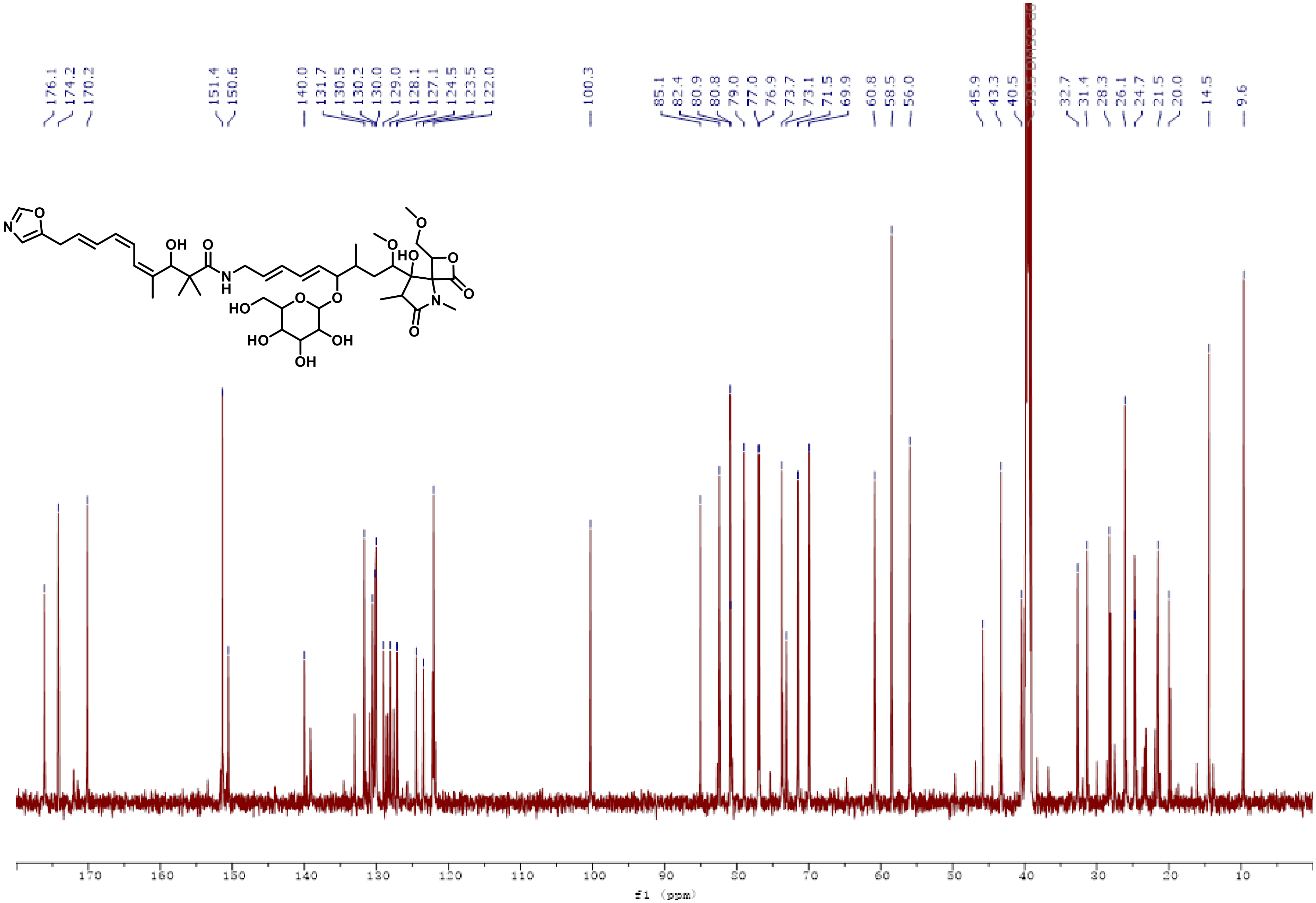

Supplementary Figure 23. ^{13}C NMR (DMSO-d_6, 700 MHz) of 7-glycosyl oxazolomycin D

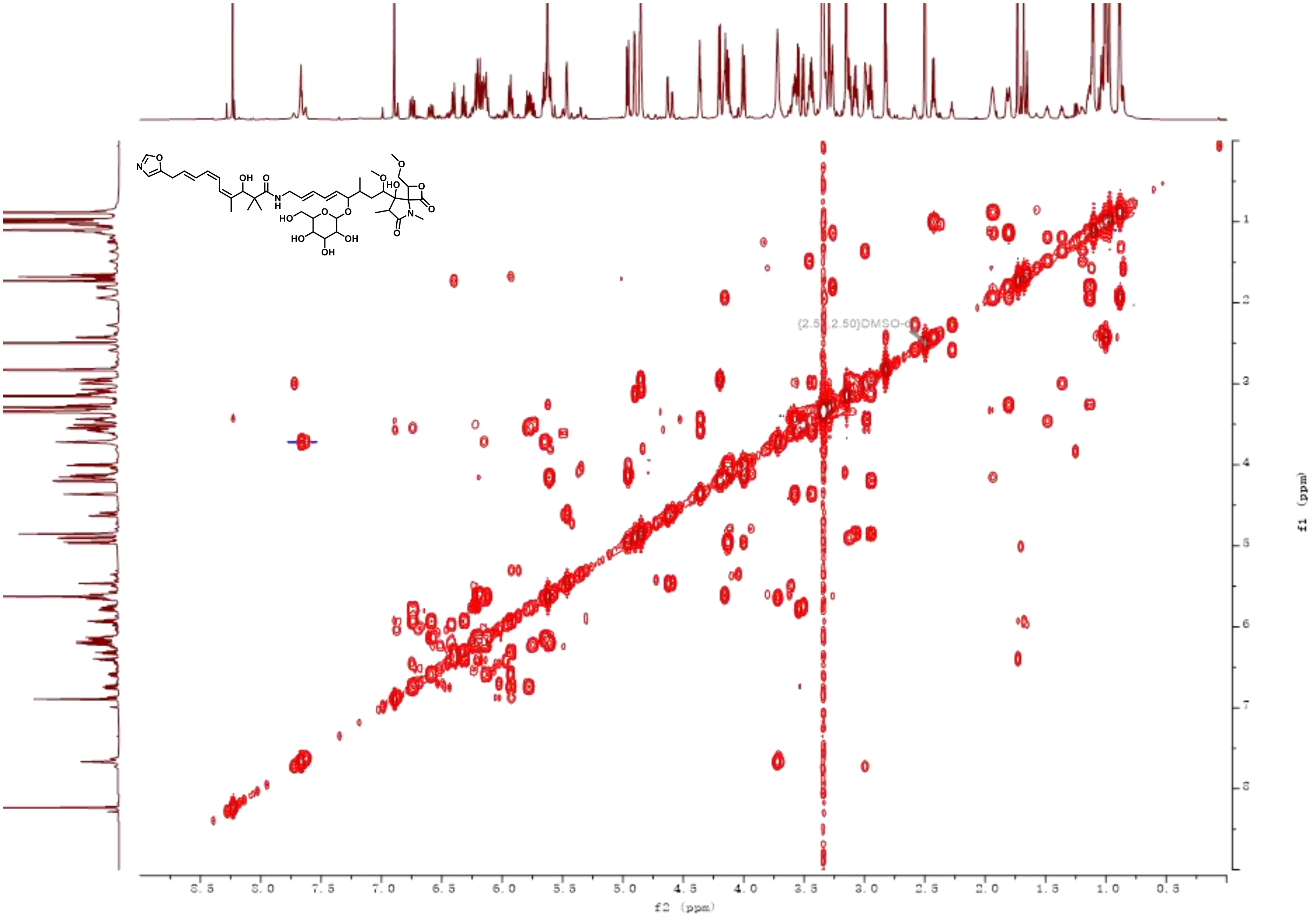

Supplementary Figure 24. COSY NMR (DMSO-d_6, 700 MHz) of 7-glycosyl oxazolomycin D

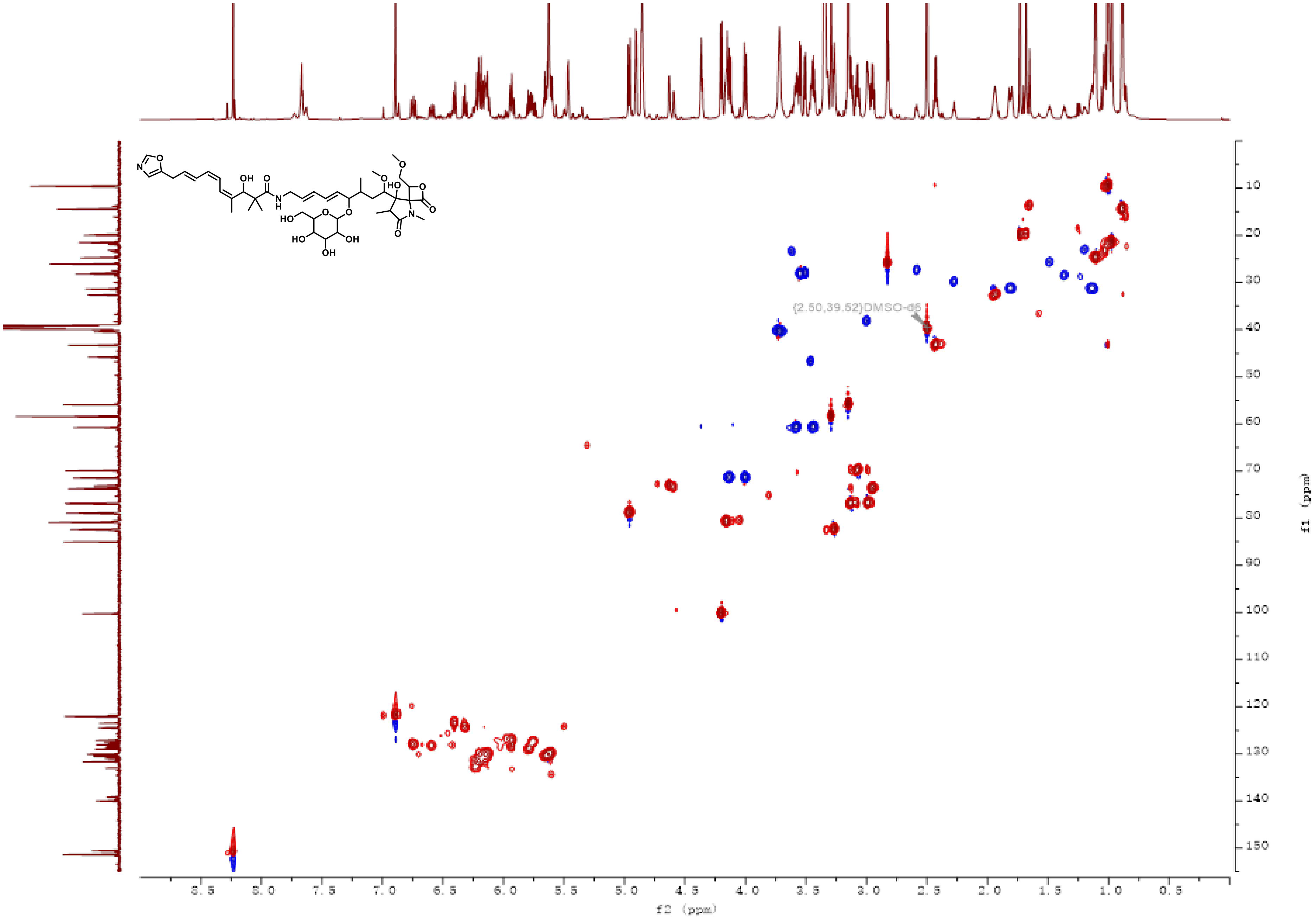

Supplementary Figure 25. HSQC NMR (DMSO-d_6, 700 MHz) of 7-glycosyl oxazolomycin D

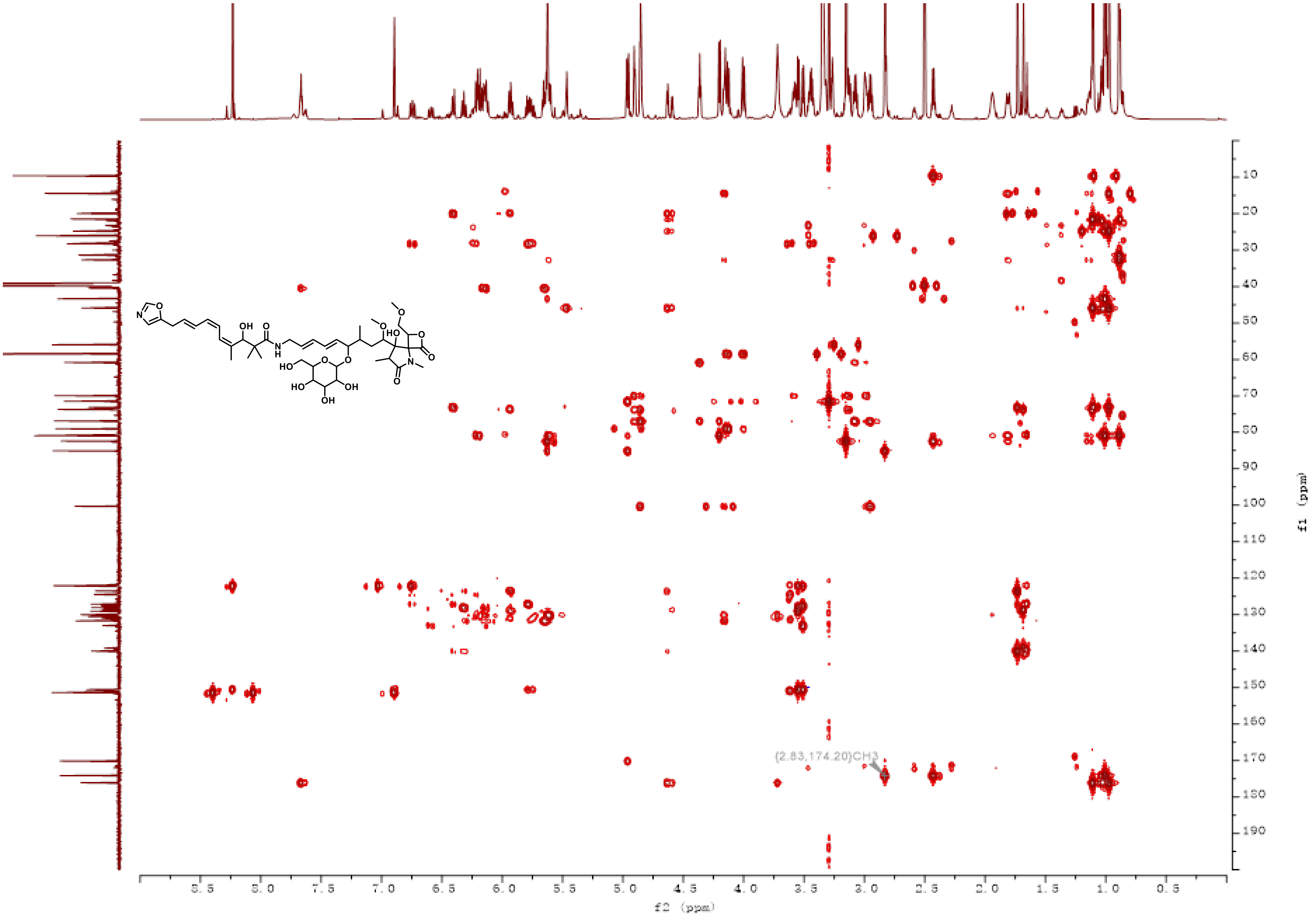

Supplementary Figure 26. HMBC NMR (DMSO-d_6, 700 MHz) of 7-glycosyl oxazolomycin D

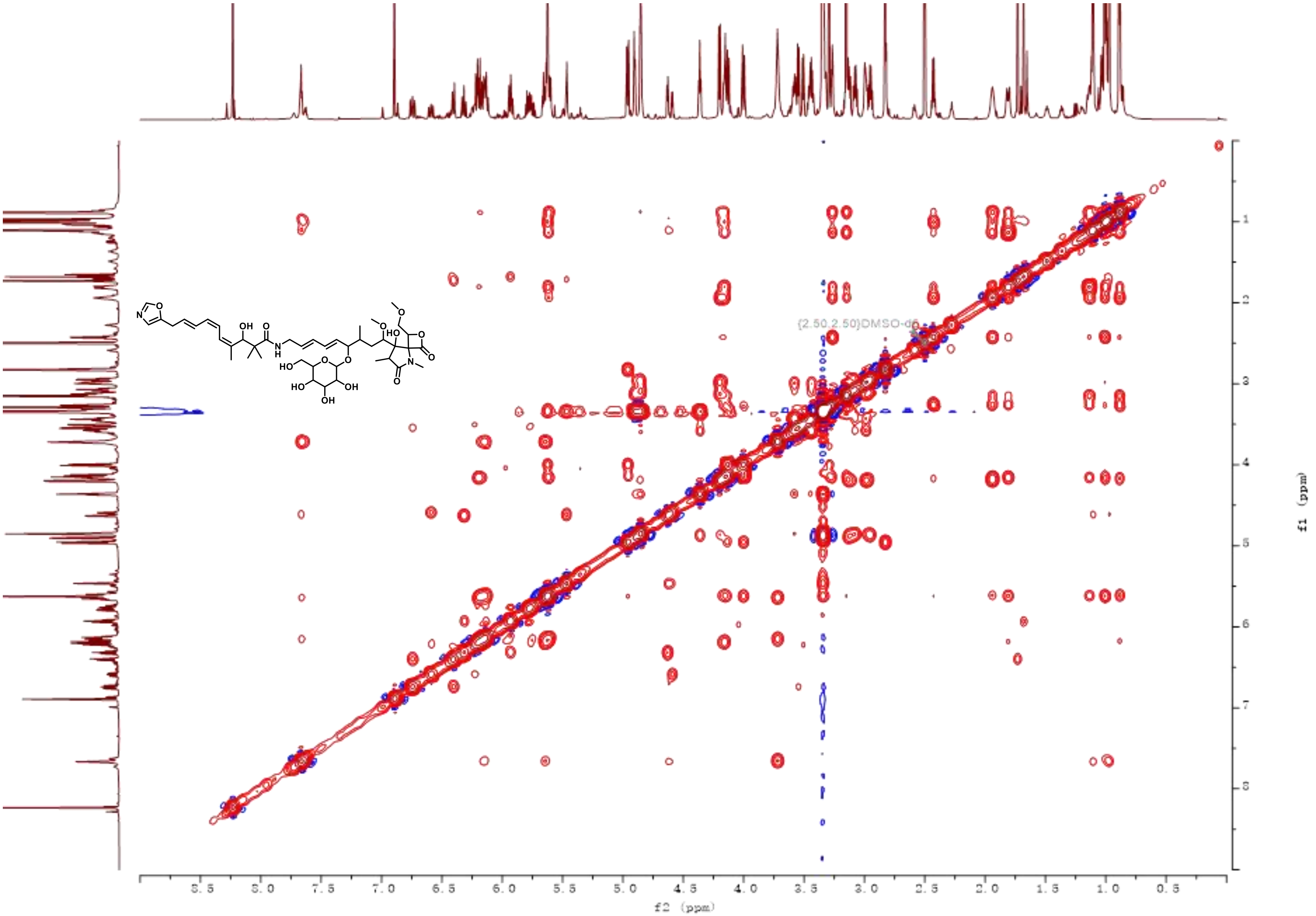

Supplementary Figure 27. NOESY NMR (DMSO-d_6, 700 MHz) of 7-glycosyl oxazolomycin D

Supplementary Table 1. NMR spectral data for 7-glycosyl oxazolomycin D in DMSO-d_6 at 700 MHz

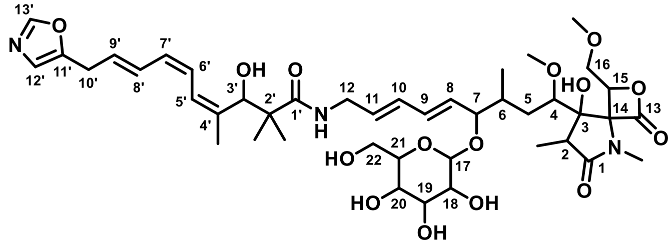

carbon #	δ_C	δ_H, mult (*J* in Hz)	COSY	HMBC
1	174.2	--	--	--
1-NMe	26.1	2.83, s	--	1,14
2	43.3	2.43, q (7.3)	2-Me	1,2-Me,3
2-Me	9.6	1.00, d (7.4)	2	1,2,3
3	80.8	--	--	--
4	82.4	3.27, t (4.9)	5a,5b	4-OMe
4-OMe	56.0	3.15, s	--	4
5a	31.4	1.81, m	4,5b	3,4,6-Me
5b		1.14, m	4,5a	3,4,6-Me
6	32.7	1.94, m	6-Me,7	--
6-Me	14.5	0.88, dd (7.0, 2.8)	6	5,6,7
7	80.9	4.16, m	6,8	6-Me,8,9,17
8	130.0	5.62, m	7,9	6,7,10
9	131.7	6.20, m	8,10	7
10	130.2	6.14, m	9,11	12
11	130.5	5.65, m	10,12	9,12
12	40.5	3.72, m	1'-NH,11	1',10
13	170.2	--	--	--
14	85.1	--	--	--
15	79.0	4.96, dd (8.9, 2.7)	16a,16b	3,13,14,16
16a	71.5	4.14, dd (12.1, 9.0)	15	15,16-OMe
16b		4.00, dd (12.3, 2.7)	15	15,16-OMe
16-OMe	58.5	3.29, s	--	16
17	100.3	4.20, d (7.6)	18	7,21
18	73.7	2.95, m	17,18-OH	17,19
18-OH	--	4,86, overlap	18	17,18,19
19	77.0	3.13, s	19-OH	18,20
19-OH	--	4.90, d (4.7)	19	18,19,20
20	69.9	3.08, m	21,20-OH	18,22
20-OH	--	4.85, overlap	20	19,20
21	76.9	2.99, m	20,22a,22b	20
22a	60.8	3.58, dd (11.9, 2.4)	21,22-OH	--
22b		3.44, m	21,22-OH	--
22-OH	--	4.36, m	22a,22b	21,22
1'	176.1	--	--	--
1'-NH	--	7.67, t (5.7)	12	1',12
2'	45.9	--	--	--
2'-Me(a)	21.5	0.97, m	--	1',2',2'-Me(b),3'
2'-Me(b)	24.7	1.10, m	--	1',2',2'-Me(a),3'
3'	73.1	4.63, d (5.3)	3'-OH	1',2',2'-Me(a)<br>2'-Me(b),4'-Me,5'
3'-OH	--	5.46, br s	3'	2'
4'	140.0	--	--	--
4'-Me	20.0	1.73, s	--	3',4',5'
5'	123.5	6.40, d (11.9)	6'	4',4'-Me,7'
6'	124.5	6.32, t (11.4)	5',7'	4',8'
7'	127.1	5.93, dd (12.5, 9.6)	6',8'	4',4'-Me,5',9'
8'	128.1	6.75, m	7',9',10'	10'
9'	129.0	5.79, m	8',10'	7',10',11'
10'	28.3	3.55, d (6.9)	9'	9',11',12'
11'	150.6	--	--	--
12'	122.0	6.89, s	--	11',13'
13'	151.4	8.23, s	--	11',12'

Supplementary Table 2 Datasets produced in this study

Dataset	Instrument	Massive ID	Zenodo link
32 mix_AQC treatment + Not treated samples	Q Exactive HF	MSV000093502	https://zenodo.org/records/10043877
University of Tuebingen NP library_AQC treatment (raw files)	Q Exactive HF	MSV000093501	https://zenodo.org/records/10043929
University of Tuebingen NP library_AQC treatment (mzml)	Q Exactive HF	MSV000093501	https://zenodo.org/records/10043937
32 mix_Cysteine treatment	QTOF Impact II	MSV000093504	https://zenodo.org/records/10459190
University of Tuebingen NP library_Cysteine treatment	QTOF Impact II	MSV000093503	https://zenodo.org/records/10459434
32 mix_Hydroxylamine treatment	QTOF Impact II	MSV000093505	https://zenodo.org/records/10459405
University of Tuebingen NP library_Hydroxylamine treatment	QTOF Impact II	MSV000093506	https://zenodo.org/records/10459447
University of Tuebingen NP library_No treatment	QTOF Impact II	MSV000093762	https://zenodo.org/records/10459457
32 mix_Not treated	QTOF Impact II	MSV000093763	https://zenodo.org/records/10461538
Untargeted_Analysis_DSM41230	QTOF Impact II	-	https://zenodo.org/records/13793599
Radical Cysteine formation	QTOF Impact II	-	https://zenodo.org/records/13794738
Cystargolide A - cysteine experiments	QTOF Impact II	-	https://zenodo.org/records/13817305
All Mzmine 4 data	-	-	https://zenodo.org/records/13819366
GNPS2 Results	-	-	https://zenodo.org/records/13969465
