# Supplementary material for: Enhancing tandem mass spectrometry-based metabolite annotation with online chemical labeling
Source: Nat Commun. 2025 Jul 26;16:6911. doi: 10.1038/s41467-025-61240-z (PMC12297239; doi:10.1038/s41467-025-61240-z)
Supplement: Supplementary file 2 — Description of Additional Supplementary Files [file 41467_2025_61240_MOESM2_ESM.pdf]

Description of Additional Supplementary Files

File Name: Supplementary Data 1

Description: General Table with all the tested NPs

File Name: Supplementary Data 2

Description: Table reactions with SMARTS
