# Supplementary material for: Enhancing tandem mass spectrometry-based metabolite annotation with online chemical labeling
Source: Nat Commun. 2025 Jul 26;16:6911. doi: 10.1038/s41467-025-61240-z (PMC12297239; doi:10.1038/s41467-025-61240-z)
Supplement: Supplementary file 5 — Reporting Summary [file 41467_2025_61240_MOESM5_ESM.pdf]

nature portfolio

Corresponding author(s): Chambers Hughes, Daniel Petras

Last updated by author(s): May 24, 2025

Reporting Summary

Nature Portfolio wishes to improve the reproducibility of the work that we publish. This form provides structure for consistency and transparency in reporting. For further information on Nature Portfolio policies, see our Editorial Policies and the Editorial Policy Checklist.

Statistics

For all statistical analyses, confirm that the following items are present in the figure legend, table legend, main text, or Methods section.

n/a	Confirmed	
☒	☐	The exact sample size (n) for each experimental group/condition, given as a discrete number and unit of measurement
☒	☐	A statement on whether measurements were taken from distinct samples or whether the same sample was measured repeatedly
☒	☐	The statistical test(s) used AND whether they are one- or two-sided<br>*Only common tests should be described solely by name; describe more complex techniques in the Methods section.*
☒	☐	A description of all covariates tested
☒	☐	A description of any assumptions or corrections, such as tests of normality and adjustment for multiple comparisons
☒	☐	A full description of the statistical parameters including central tendency (e.g. means) or other basic estimates (e.g. regression coefficient) AND variation (e.g. standard deviation) or associated estimates of uncertainty (e.g. confidence intervals)
☒	☐	For null hypothesis testing, the test statistic (e.g. F, t, r) with confidence intervals, effect sizes, degrees of freedom and P value noted<br>*Give P values as exact values whenever suitable.*
☒	☐	For Bayesian analysis, information on the choice of priors and Markov chain Monte Carlo settings
☒	☐	For hierarchical and complex designs, identification of the appropriate level for tests and full reporting of outcomes
☒	☐	Estimates of effect sizes (e.g. Cohen's d, Pearson's r), indicating how they were calculated

Our web collection on statistics for biologists contains articles on many of the points above.

Software and code

Policy information about availability of computer code

Data collection	Xcalibur (4.1), Data Analysis, SIRIUS 6, mzmine 4, GNPS2
Data analysis	The code for the GNPS workflow and analysis is available on GitHub: https://github.com/Wang-Bioinformatics-Lab/Chemical_Metabolomics_with_functional_groups/tree/master and Zenodo https://zenodo.org/records/15469092. A fully runnable instance of the workflow can be launched directly at https://gnps2.org/workflowinput?workflowname=Chemical_Metabolomics_With_Functional_Groups. The mzmine source code and versions are available on GitHub https://github.com/mzmine/mzmine. MCheM filtering is available in SIRIUS version 6.2, and source code and versions are available on GitHub https://github.com/sirius-ms/sirius. The code for filtering SIRIUS candidate lists via MCheM can be found at https://github.com/kaibioinfo/functional_metabolomics. The code for the SMARTS check is uploaded to https://github.com/corinnabrungs/smarts_testing and zenodo https://doi.org/10.5281/zenodo.15480298.

For manuscripts utilizing custom algorithms or software that are central to the research but not yet described in published literature, software must be made available to editors and reviewers. We strongly encourage code deposition in a community repository (e.g. GitHub). See the Nature Portfolio guidelines for submitting code & software for further information.

Data

Policy information about availability of data

All manuscripts must include a data availability statement. This statement should provide the following information, where applicable:

- Accession codes, unique identifiers, or web links for publicly available datasets
- A description of any restrictions on data availability
- For clinical datasets or third party data, please ensure that the statement adheres to our policy

All MS data acquired in this work are publicly accessible both through MASSIVE with the following identifiers: MSV000093502, MSV000093501, MSV000093504, MSV000093503, MSV000093505, MSV000093506, MSV000093762, MSV000093763. Additionally, all raw data as well as processed data, and batch files, are publicly available through Zenodo. All links are listed in the supplemental information in Supplementary Table 7. The CANOPUS dataset used to test the method can be downloaded from https://bio.informatik.uni-jena.de/data/. Streptomyces libani subsp. rufus DSM 41230 (strain NBRC 15424) genome is publicly accessible on https://www.ncbi.nlm.nih.gov/ with accession number AP023408. S. libani DSM 41230 GNPS2 FBMN job is publicly available at the following link: https://gnps2.org/status?task=714b3ae18f8d4c499876d486658b9b30. GNPS2 Chemical Metabolomics job for the experimental dataset can be accessed here: https://gnps2.org/status?task=508a9676780d4e119cb6ac3bce011512. NMR data has been deposited to nmrXiv (www.nmrxiv.org) with the following DOI: 10.57992/nmrxiv.p114. Source data for Figure 3 is available in the Supplementary Information.

Research involving human participants, their data, or biological material

Policy information about studies with human participants or human data. See also policy information about sex, gender (identity/presentation), and sexual orientation and race, ethnicity and racism.

Reporting on sex and gender	Not applicable
Reporting on race, ethnicity, or other socially relevant groupings	Not applicable
Population characteristics	Not applicable
Recruitment	Not applicable
Ethics oversight	Not applicable

Note that full information on the approval of the study protocol must also be provided in the manuscript.

Field-specific reporting

Please select the one below that is the best fit for your research. If you are not sure, read the appropriate sections before making your selection.

[x] Life sciences [ ] Behavioural & social sciences [ ] Ecological, evolutionary & environmental sciences

For a reference copy of the document with all sections, see nature.com/documents/nr-reporting-summary-flat.pdf

Life sciences study design

All studies must disclose on these points even when the disclosure is negative.

Sample size	The study includes only a set of authentic standards for method validation and a biological sample as a case study.
Data exclusions	Not applicable
Replication	Samples were analyzed in technical duplicates with and without MCheM.
Randomization	For the analysis of each sample, 5 µL were injected and analyzed in duplicates with and without MCheM procedure for each derivatization. The sample order was randomized within the same batch.
Blinding	Not applicable

Reporting for specific materials, systems and methods

We require information from authors about some types of materials, experimental systems and methods used in many studies. Here, indicate whether each material, system or method listed is relevant to your study. If you are not sure if a list item applies to your research, read the appropriate section before selecting a response.

Materials & experimental systems

n/a	Involved in the study
☒	☐ Antibodies
☒	☐ Eukaryotic cell lines
☒	☐ Palaeontology and archaeology
☒	☐ Animals and other organisms
☒	☐ Clinical data
☒	☐ Dual use research of concern
☒	☐ Plants

Methods

n/a	Involved in the study
☒	☐ ChIP-seq
☒	☐ Flow cytometry
☒	☐ MRI-based neuroimaging

Plants

Seed stocks	Not applicable
Novel plant genotypes	Not applicable
Authentication	Not applicable
